# Nighttime road scene image enhancement based on cycle-consistent generative adversarial network

**DOI:** 10.1038/s41598-024-65270-3

**Published:** 2024-06-22

**Authors:** Yanfei Jia, Wenshuo Yu, Guangda Chen, Liquan Zhao

**Affiliations:** 1 College of Electrical and Information Engineering, Beihua University, Jilin, 132013 China; 2 College of Electrical Engineering, Northeast Electric Power University, Jilin, 132012 China

**Keywords:** Generative adversarial network, Nighttime road scene image enhancement, Encoder-decoder netwrok, Illumination attention module, Engineering, Mathematics and computing

## Abstract

During nighttime road scenes, images are often affected by contrast distortion, loss of detailed information, and a significant amount of noise. These factors can negatively impact the accuracy of segmentation and object detection in nighttime road scenes. A cycle-consistent generative adversarial network has been proposed to address this issue to improve the quality of nighttime road scene images. The network includes two generative networks with identical structures and two adversarial networks with identical structures. The generative network comprises an encoder network and a corresponding decoder network. A context feature extraction module is designed as the foundational element of the encoder-decoder network to capture more contextual semantic information with different receptive fields. A receptive field residual module is also designed to increase the receptive field in the encoder network.The illumination attention module is inserted between the encoder and decoder to transfer critical features extracted by the encoder to the decoder. The network also includes a multiscale discriminative network to discriminate better whether the image is a real high-quality or generated image. Additionally, an improved loss function is proposed to enhance the efficacy of image enhancement. Compared to state-of-the-art methods, the proposed approach achieves the highest performance in enhancing nighttime images, making them clearer and more natural.

## Introduction

The low-light conditions at night make it challenging for machine vision algorithms to complete tasks, such as image segmentation and target recognition, due to the unclear images produced by image sensors^1^. With the development of artificial intelligence, some nighttime road scene image enhancement methods based on artificial intelligence are proposed^2^. Although artificial intelligence-based image enhancement methods can improve the clarity of nighttime images, enhanced images still exhibit considerable noise, significant contrast, and detail distortion issues. Therefore, we propose an innovative cycle-consistent generative adversarial network framework to reduce noise and distortion of enhanced nighttime road scene images. The network is trained with unpaired datasets and no longer depends on the single mapping of the same scene to be adaptive to various complex environments. The network consists of four parts: forward generative network, inverse generative network, forward discriminative network, and inverse discriminative network. This approach involves using two types of generative networks: a forward generative network and an inverse generative network. The forward generative network generates high-quality images of road scenes, while the inverse generative network generates lower-quality images. Additionally, a discriminative network is used to determine whether an input image is original or has been enhanced by the generative network. The interplay between adversarial and generative networks refines the generative networks’ image enhancement capabilities through training. We have designed a new type of generative adversarial network that can enhance images of nighttime road scenes. This network includes two generative and two discriminative networks that share the same structures.

The main contributions of this research are as follows:

1. A generative network is designed with an encoder-decoder architecture. To improve its performance, a context feature extraction module is designed. This module is excellent at capturing contextual semantic details across a range of receptive field sizes and has been integrated into both the encoder and decoder networks. An illumination attention module is also designed to transmit important features of different depth networks in the encoder network to the decoder network. A receptive field residual module has been developed to increase the receptive field of the encoder. Additionally, the Resnet-D module has been used as a replacement for the downsampling operation to preserve crucial feature information.
2. A multiscale discriminative network has been developed to improve the network’s ability to accurately distinguish between the original and generated images. To further enhance its performance two skip connections have been incorporated into the network to improve its performance further. These connections help to combine low-frequency and high-frequency information from the feature map, thereby ensuring that crucial is not lost. This fusion process contributes to improving the effectiveness of the discriminative network.
3. We have developed an improved loss function that combines the least squares loss and unsupervised perceptual loss with the conventional loss function. This combination provides a more comprehensive evaluation of the abilities of both generative and discriminative networks. Using the least squares loss function serves two purposes: it helps avoid issues related to gradient vanishing and effectively reduces the gap between sample features and the decision boundary. This alignment significantly contributes to the improvement of image quality.

## Related work

The approaches for enhancing nighttime road scene images include techniques based on histogram equalization^3^, those based on the Retinex theory^4^, strategies based on deep learning^5^ and their hybrid methods such as Retinex-based deep unfolding network^6^ and histogram equalization multiscale Retinex combination approach^7^. Histogram equalization-based techniques concentrate on reshaping an image’s histogram distribution to achieve a uniform distribution. This manipulation serves to amplify the range of gray value disparities among pixels, effectively enhancing image contrast. Consequently, such methods are predominantly employed to augment the contrast of images characterized by limited dynamic ranges^8^. For example, Singh et al. introduced a recursive histogram equalization strategy for image enhancement^9^. This technique partitions the image histograms, performs histogram division based on distinct exposure thresholds, and subsequently equalizes each partition’s histogram. Although this approach effectively tackles the issue of enhancing low-exposure images, it falls short of effectively mitigating the influence of image noise. In contrast, Chen et al. introduced a contrast enhancement technique based on the entropy-preserving mapping prior^10^. This method aims to restore image contrast and texture by formulating a closed-form solution for image contrast under the specified prior condition. The coefficients of this solution are learned through unsupervised learning. However, obtaining accurate priors for this approach proves challenging, as inaccuracies in the priors can lead to undesirable artifacts in the enhanced image.

The enhancement approach for nighttime road scene images, rooted in the Retinex theory, decomposes the image into an illumination component and a reflection component. The reflection component is maintained constant, while the illumination component’s brightness is heightened, ultimately leading to image restructuring^11^. Ren et al. introduced a solution to the image enhancement problem through a low-rank regularized Retinex model^12^. By incorporating a low-rank prior into the Retinex decomposition process, this technique effectively eliminates noise in the reflection component of the image. Nonetheless, this method suffers from color distortion. Another strategy, proposed by Zhang et al., leverages double illumination estimation to enhance images^13^. This method involves obtaining two exposure correction images via double illumination estimation of the input image. These images are then fused using an image fusion technique, merging the visually most effective exposure portion of the input image to yield a globally well-exposed output. While this technique primarily addresses mis-exposure, it exclusively considers illumination effects, disregarding the impact of noise. Hao et al. introduced a semi-decoupled image enhancement method rooted in the Retinex framework^14^. This method undertakes gradual estimation of the illumination component while conducting a joint analysis of the reflection component and the intermediate layer. Notably, it efficiently suppresses noise within the reflection component, resulting in enhanced images of superior visual quality. However, due to its reliance on reflection components, this method’s applicability is restricted, and its generalization capability across diverse, complex environments is limited. Hybrid Retinex and deep learning methods still have the same limitations as conventional Retinex-based methods, such as ideal assumption^15^.

In the swiftly advancing landscape of artificial intelligence, the integration of deep learning has revolutionized nighttime road scene image enhancement^16^. The methodology anchored in deep learning involves training models comprising deep convolutional neural networks with image data from a training set. The objective is to establish a mapping correlation between images of low quality and high quality, thereby significantly enhancing the overall quality of nighttime road scene images. Compared to techniques rooted in histogram equalization and the Retinex theory, deep learning-based approaches exhibit superior image quality and a broader range of applicability^17^. For example, Li et al. introduced a new convolutional neural network specifically designed for enhancing low-light images^18^. The network takes in a low-quality image, producing an improved illumination component map. This map is subsequently used within a Retinex-based model to obtain high-quality images. However, this method exhibits the checkerboard artifact when dealing with noisy low-light images. To tackle this challenge, Cai et al. introduced a single-image contrast enhancement technique that effectively handles both overexposure and underexposure scenarios^19^. This method employs three convolutional neural network-based enhancement networks to manage image contrast enhancement, detail recovery, and harmonizing detail and texture. Nevertheless, this approach might lead to overexposure issues.

Ren et al. designed a deep hybrid network specifically for improving image quality^20^. Through an encoder-decoder architecture, this approach gauges the overall content of input images. Furthermore, it introduces an innovative spatially variant recurrent neural network to capture intricate details. Although it excels in enhancing degraded images, this method disregards local information, potentially losing details within the improved output. Wang et al. presented the DeepUPE network for image enhancement^21^. This network establishes an image-to-illumination mapping relationship and enhances low-exposure images using illumination mapping. However, it overlooks the noise present in the original image. Fan et al. introduced an image enhancement technique incorporating semantic segmentation and the Retinex model^22^. This technique significantly enhances image enhancement by incorporating prior information derived from image semantics to direct the enhancement of both the illumination and reflection components. However, it exhibits vulnerability to overexposure problems. In response, Guo et al. devised an image enhancement approach named Zero-DCE (Zero-Reference Deep Curve Estimation)^23^. This approach employs a neural network to approximate the illumination mapping curve, making it particularly beneficial for adjusting the image’s dynamic range. Yet, it tends to exhibit overexposure problems. In response to these challenges, Li et al. introduced a lightweight deep curve estimation method named Zero-DCE++^15^. This optimization of Zero-DCE streamlines the network structure and inference speed by simplifying the illumination enhancement curve. However, overexposure concerns remain unresolved.

Generative Adversarial Networks (GANs) constitute a distinct category of deep learning models known for their capability to enhance model performance through a competitive interplay between the generative and discriminative networks^24,25^. GANs find extensive utility in diverse image processing domains, encompassing image generation, dehazing, super-resolution, and more^26,27^. GANs also demonstrate potential for nighttime road scene image enhancement within this broad context. Bose et al. introduced an innovative framework named LumiNet for enhancing backlit images^28^, leveraging a modified U-Net in conjunction with a unique discriminator-based conditional generative adversarial network. This approach successfully achieved a harmonious balance in exposure levels between foreground and background regions. Tao et al. designed a novel basic residual block. They utilized the designed block to construct a generative adversarial network to enhance low-light images within the industrial Internet of Things (IoT) intelligent camera system. They also designed the Harr down-sampling layer to separate high and low-frequency signals^29^. Son et al. proposed to utilize the Stevens effect and local blur map to process the enhanced night road images by the cycle-consistent generative adversarial network to reduce the noise and enhance detail information^30^. Chen et al. proposed an improved generative adversarial network to enhance the image quality of nighttime images and rain images by introducing the attention mechanism modules and the multiscale feature fusion modules into the generator network and local discrimination strategy into the discriminator^31^. Zhang et al. designed a Decompose-Enhance-GAN Network tailored for augmenting low-light images^32^. They partitioned the input low-light image into reflectance and illumination components, facilitating Enhance-Net’s efficient enhancement of the illumination aspect. Zhou et al. also applied the style transfer of cycle-consistent generative adversarial networks to low illumination image enhancement^33^. Fu et al. proposed an unsupervised low-light image enhancement method based on generative adversarial networks named LE-GAN^34^. This method addressed the challenges of noise, color bias, and over-exposure without paired training data. The network incorporated an illumination-aware attention module, enhancing feature extraction to mitigate noise and color bias while improving visual quality. However, the structure of the LE-GAN is rather complex, thus requiring more computational resources and time during the training process, as well as higher-performance hardware for training. Jiang et al. introduced an image enhancement approach, “EnlightenGAN,” grounded in generative adversarial networks^35^. This approach avoids relying on paired images and establishes an unpaired map-ping between low-light and normal-light images. However, due to its exclusive focus on illumination effects while neglecting noise interference, the enhanced images it produces are prone to artifacts. In a similar vein, Ni et al. proposed a novel unsupervised image-enhanced generative adversarial network^36^. This technique incorporates a global attention module to capture overarching image features and introduces fidelity loss to preserve intricate details. While this approach yields better image quality improvements, the enhanced images often still contain artifacts and noise. Furthermore, many studies also have reviewed the application of GANs in enhancing low-light images^37,38^.

Traditional methods for enhancing nighttime road scene images, such as histogram equalization, have certain limitations. For example, they may lead to over-enhancement or parts of the image that are not sufficiently enhanced, resulting in an unnatural or unrealistic appearance. Moreover, these techniques might not be effective for images with complex lighting conditions or a high dynamic range. Retinex-based methods for enhancing nighttime road scene images can also have drawbacks. These techniques rely on the presumption that the image may be broken down into components for reflection and illumination. However, this assumption may not always hold in practical scenarios, and the resulting output may not always be visually pleasing or realistic. Deep learning methods are preferred for enhancing nighttime road scene images because they can automatically learn complex nonlinear mappings between input and output images. These methods can automatically learn relevant features from the input image and utilize them to improve the image in a data-driven manner. Furthermore, deep learning methods can handle images with complex lighting conditions and high dynamic range, which are generally challenging for traditional methods. Another benefit of deep learning methods is their ability to generalize well to unseen data, which makes them suitable for a wide range of applications. These methods can also be trained on large datasets and optimized for specific performance metrics, leading to improved accuracy and robustness. Compared with conventional deep learning-based road scene image enhancement methods, while GAN-based approaches yield superior image quality enhancements, there remains a noticeable gap between the enhanced and ideal images. This gap is particularly evident in significant noise, overexposed or underexposed regions, thereby detrimentally impacting image quality. We present a new cycle-consistent generative adversarial network to enhance the quality of images. Our tests confirm the efficacy of this network in improving the image’s quality.

### Proposed cycle-consistent generative adversarial network

**Figure 1:**
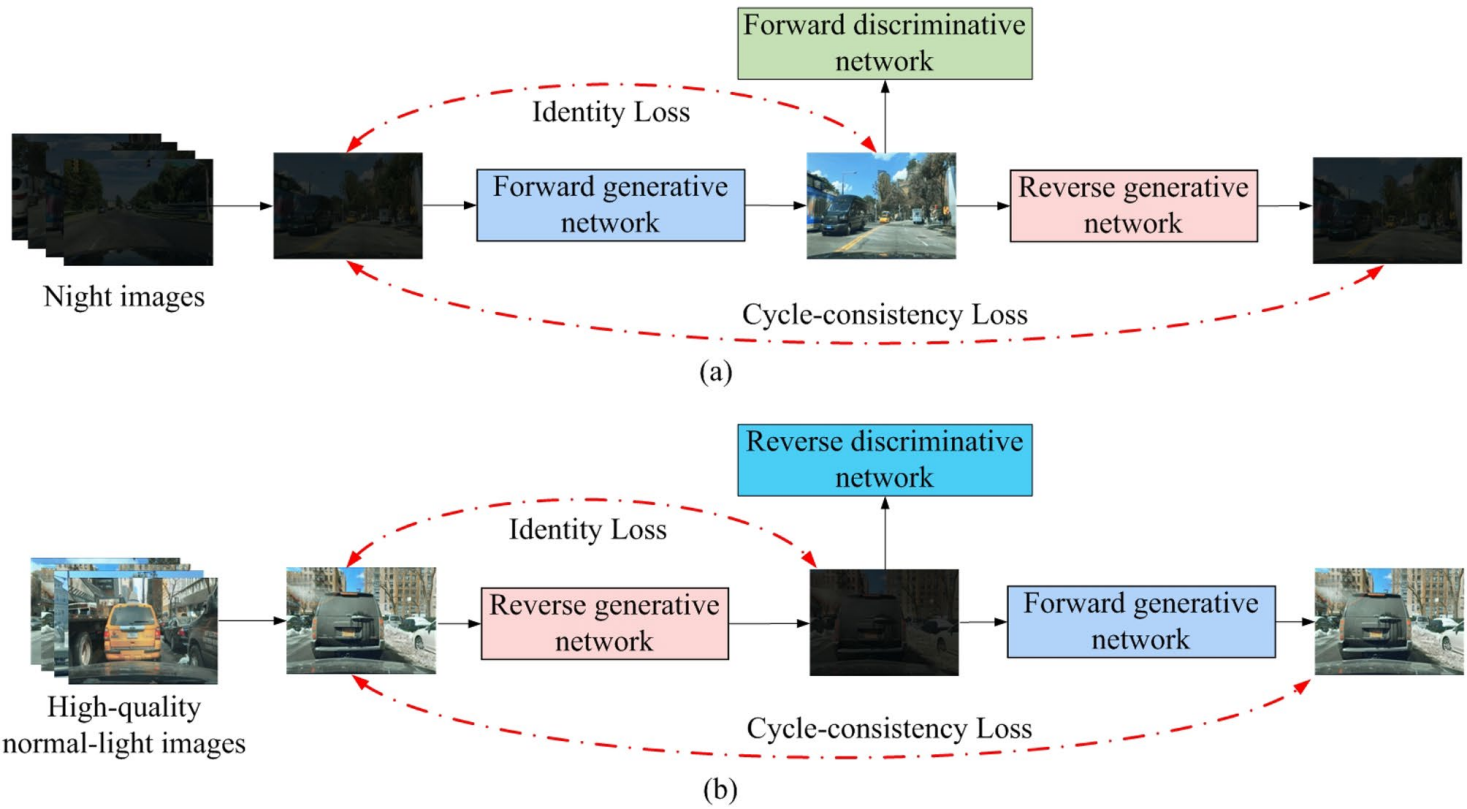
The framework of the cycle-consistent generative adversarial network. (**a**) represents the forward network framework, and (**b**) represents the reverse network framework.

We design a generative adversarial network that enhances nighttime road scene images with cycle consistency. This network is designed to improve the image quality of autonomous vehicles during nighttime road scenes. The cycle-consistent generative adversarial network consists of four main components: the forward and reverse generative networks and the forward and reverse discriminative networks. The forward and reverse generative networks have distinct roles. The former enhances nighttime road scene images, while the latter generates nighttime road scene images with reduced illumination. The forward discriminative network distinguishes between input images representing improved nighttime road scenes and real images illuminated under normal conditions. On the other hand, the reverse discriminative network is responsible for identifying whether input images are generated at nighttime road scenes or actual low-illumination images taken at night. Figure 1 provides a diagrammatical representation of the cycle-consistent generative adversarial network. It’s noteworthy that the forward and reverse generative networks have the same architecture, just like the forward and reverse discriminative networks share a standard structure. As a result, we will discuss the forward generative and forward discriminative networks in the following sections.

#### Proposed generative network

**Figure 2:**
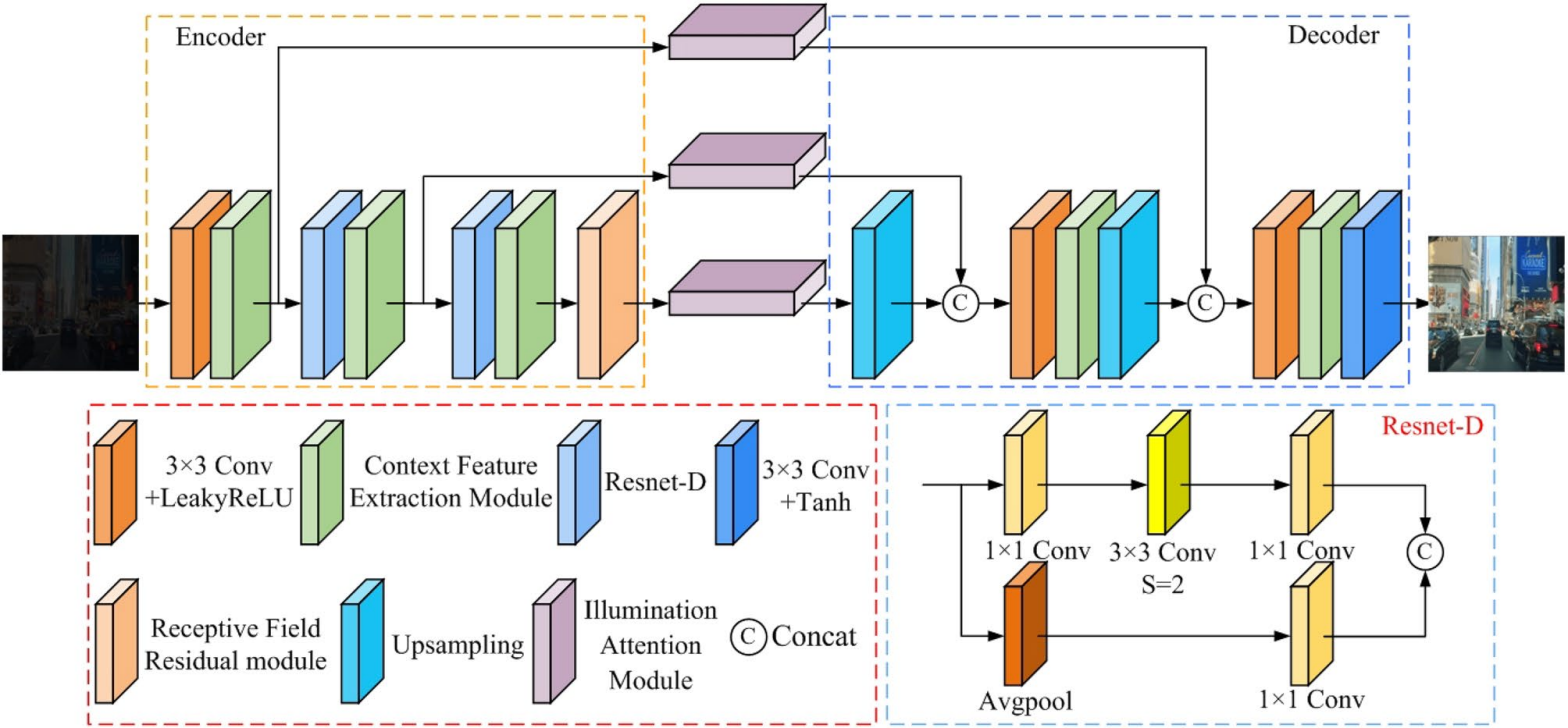
Our proposed forward generative network structure.

**Figure 3:**
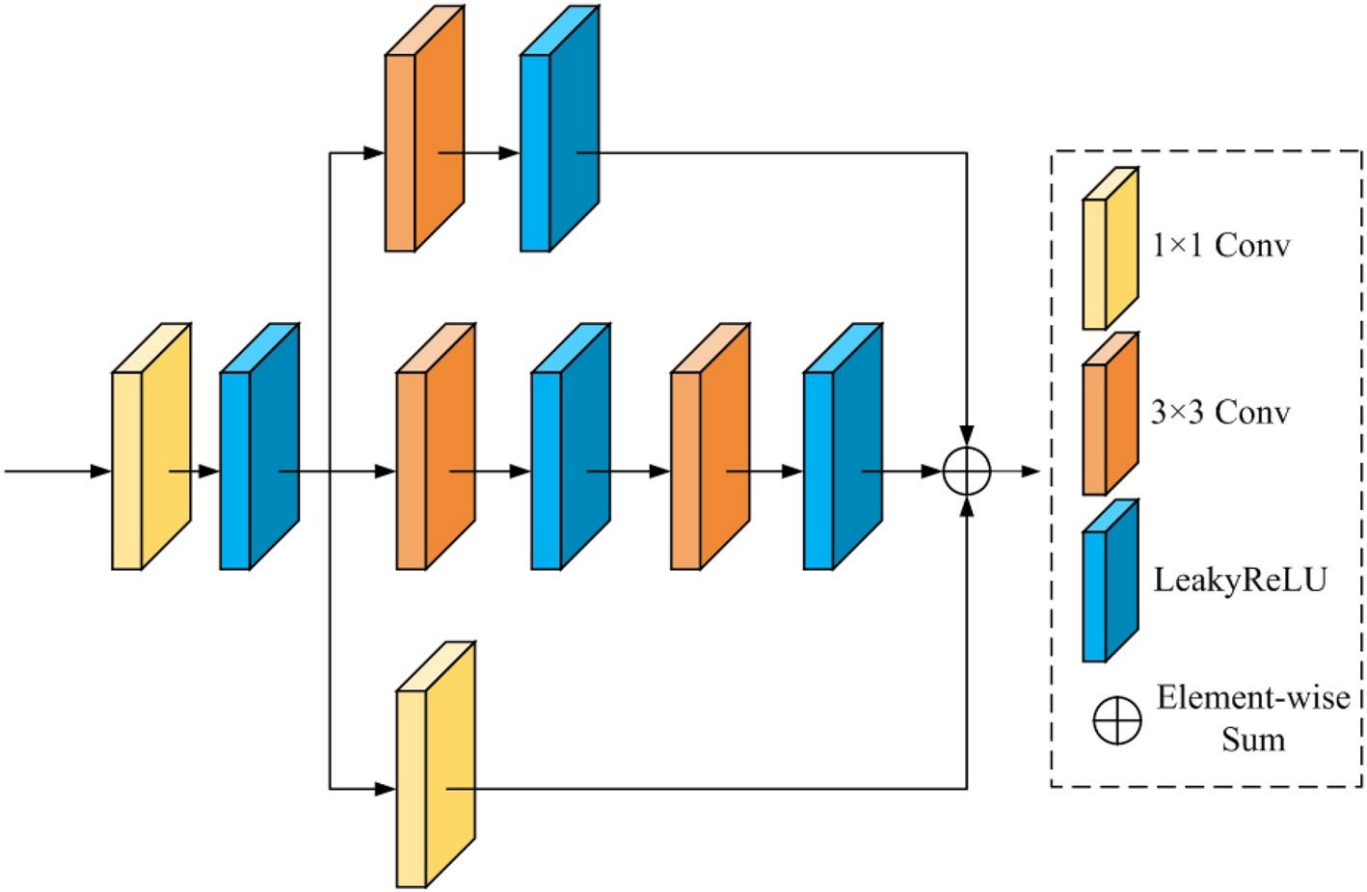
Our proposed context feature extraction module.

Our proposed network comprises a forward and reverse generative network with identical structures. In this section, we will focus solely on explaining the architecture of the forward generative network. The conventional U-Net architecture of a generative network includes an encoder and a decoder. The typical encoder comprises convolution and downsampling layers, while the decoder consists of convolution and upsampling layers. To extract more contextual semantic information, We designed context feature modules and used them to replace the convolution modules in both the encoder and decoder. To extract more global information, we design a receptive field residual module and place it in the last layer of the encoder. Additionally, the Resnet-D module has been used as a replacement for the downsampling operation to preserve crucial feature information. In the conventional U-Net structure of a generative network, the skip connections are used to fuse the features of the encoder and decoder to avoid losing information. We design illumination attention modules to extract effective illumination features and use them instead of the skip connection. The proposed forward generative network shown in Fig. 2 consists of four main components: an encoder network, a decoder network, three illumination attention modules, and a receptive field residua module. The encoder network plays a vital role in expanding the network’s receptive field by reducing the feature map’s dimensions, allowing for more effective low-frequency information extraction. On the other hand, the decoder network facilitates the extraction of high-frequency details by expanding the feature map. The last layer of the decoder network is responsible for restoring the R B image. A crucial component of this network is the illumination attention module, designed to transmit important characteristics from the encoder network to the decoder network. This allows for efficient information propagation throughout the network. Additionally, integrating a receptive field residual module at the end of the decoder network expands the network’s receptive field. This improvement significantly enhances the network’s ability to incorporate a wide range of features, extracting richer features. Recognizing the benefits of a sizable receptive field for extracting global image features and a smaller one for capturing finer details, we introduce a context feature extraction module shown in Fig. 3. The proposed context feature extraction module integrates multiple branches, each comprising convolutional layers with distinct kernel sizes, facilitating effective feature extraction across diverse receptive fields. This enables the generation network to incorporate global and local information during image enhancement, consequently enhancing the overall effectiveness of the enhancement process. As a result, this module can effectively acquire a range of receptive field sizes. Leveraging this context feature extraction module as a sub-module, we construct both the encoder and decoder networks.

The encoder network has distinct components arranged to facilitate efficient feature extraction. It commences with a convolution layer employing the LeakyReLU function, succeeded by a context feature extraction module and two consecutive cascade modules. Each cascade module includes a Resnet-D module and a context feature extraction module, as depicted in Fig. 3. This Resnet-D module, shown in Fig. 2, is characterized by two branches: one integrates three convolutions employing varied kernel sizes. At the same time, the other incorporates average pooling and convolution layers. The initial convolutional layer, paired with the LeakyReLU activation function, fulfills the role of preliminary feature extraction within the encoder network. This layer effectively elevates the channel count from 3 to 32, establishing a foundation for subsequent processing. Three context feature extraction modules are used in tandem to extract features from different-sized feature maps. Additionally, the Resnet-D module facilitates a downsampling operation that reduces the feature map’s dimensions. This operation culminates in an output feature map size half that of the input feature map for the Resnet-D module. Crucially, the resulting feature map from the context feature extraction module showcases a doubled channel count compared to the input feature map, enhancing the information’s richness.

**Figure 4:**
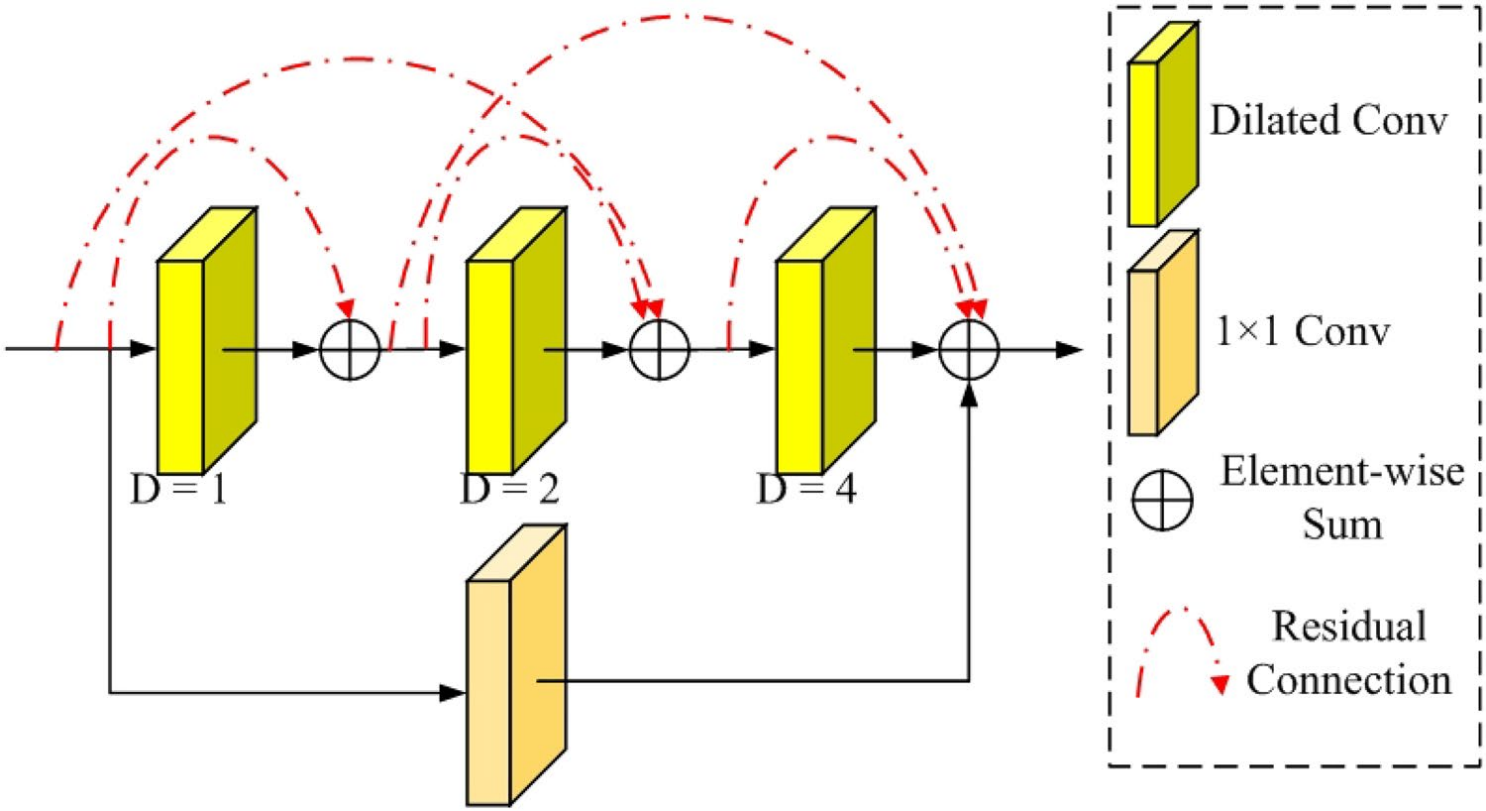
Our proposed receptive field residual module.

To further optimize the effectiveness of image enhancement, we have devised a receptive field residual module, which is illustrated in Fig. 4. It can expand the receptive field while extracting a broader range of contextual feature information. The receptive field residual module consists of three multi-level residual modules connected by the dilation convolution with 1, 2, and 4 dilation rates. The module can effectively capture low-frequency information from the feature map by efficiently enlarging the receptive field without requiring a surge in network parameters. The output feature map from the encoder network is the input feature map for the receptive field residua module. In dilated convolutions, the dilation rate refers to the spacing between values in the th kernel. Dilation rates 1, 2, and 4 are commonly chosen because they allow the network to aggregate information at different scales. A dilation rate of 1 in dilated convolutions operates on the pixel neighborhood, capturing fine details of the feature map. A dilation rate 2 enables the convolution to gather information from a broader context, obtaining feature information from a slightly larger pixel neighborhood. Finally, a dilation rate of 4 further expands the receptive field, providing more contextual information in the feature map. However, more significant dilation rates can expand the receptive field but can also lead to excessive information loss. In low-light conditions, the images already lack fine details and clarity, so further enlarging the receptive field may result in blurriness and detail loss. Additionally, in low-light conditions, excessively large receptive fields may capture unrelated contextual information, which may not be conducive to the image enhancement task. The appropriate receptive field size should focus on capturing information relevant to image enhancement.

**Figure 5:**
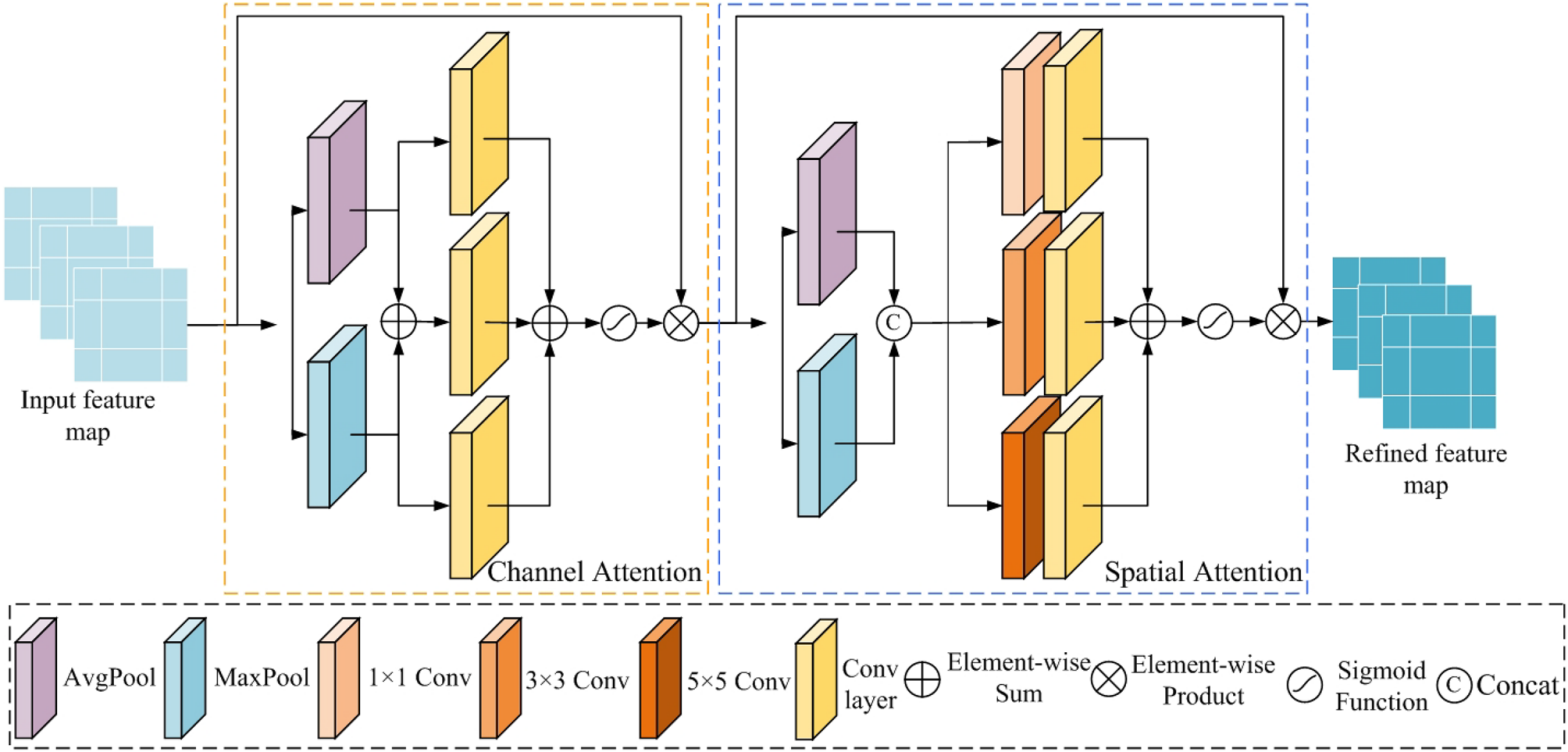
Our proposed illumination attention module.

In the decoder, we use upsampling, standard convolution with LeakyReLU activation function, and context feature extraction module to extract high-frequency feature information. Upsampling is used to increase the size of the feature map, making it twice the original size. Standard convolution and the LeakyReLU activation function are utilized to extract features and the context feature extraction module. Finally, the decoder uses a convolution layer with the Tanh activation function to reconstruct the high-quality road scene RGB image from the extracted features. We have introduced an illumination attention module to enhance the quality of images and eliminate the impact of unnecessary illumination features extracted by the encoder system. We have effectively transmitted important low-frequency features to the decoder network by employing three such modules. The structure of the illumination attention module is depicted in Fig. 5. By incorporating the Illumination Attention Module, different processing strategies, such as pooling and convolution operations, can be applied based on the characteristics of images under varying lighting conditions. This enables enhancement of the overall brightness of the images while avoiding excessive enhancement, thereby improving visual effects and enhancing image enhancement performance. The illumination attention module encompasses a channel attention module and a spatial attention module. The designed channel attention module features an assortment of components: an average pooling layer, a maximum pooling layer, and three parallel convolution layers. The average pooling layer is integrated to aggregate background information from the feature map, while the maximum pooling layer effectively gathers texture-related details. These pooling mechanisms facilitate the extraction of distinct image characteristics, contributing to a comprehensive feature representation. Incorporating parallel convolutions further enhances feature extraction. This amalgamation bolsters the network’s capability to capture intricate image attributes while promoting a holistic understanding of the input. The spatial attention module we designed is employed to alleviate the issue of underexposure and overexposure in low-light images. The spatial attention module consists of an average pooling layer, a maximum pooling layer, three parallel convolutions with different kernel sizes, and three parallel $1\times 1$ convolutions. In the spatial attention module, the maximum pooling and average pooling are used to aggregate texture and background information in the channel dimension, respectively. The aggregate information of maximum pooling and average pooling is fused by concatenation operation. Since complex image features may become more pronounced under low-light conditions, the $1\times 1$ convolution, $3\times 3$ convolution, and $5\times 5$ convolution are used to capture this information. The Sigmoid activation function is employed to obtain attention weights. At this stage, regions heavily impacted by low-light conditions are assigned larger weights, while others are assigned smaller weights or remain unchanged. This process helps to enhance the overall brightness of the dark areas of the image as well as prevent the bright areas from being over-enhanced so that the spatial attention module we designed effectively solves the underexposure and overexposure problems common to low-light image enhancement tasks. Compared to traditional attention modules, the illumination attention module we propose has more parameters.

Within the designed illumination attention module, both the channel attention and spatial attention modules can heighten the prominence of vital features in their respective channel and spatial domain. It can reduce the interference of redundant feature information and the loss of detailed feature information. The expression for the result from the illumination attention module is:

1 $$\begin{aligned} F(x) = S[C(x)] \end{aligned}$$

where *x* is the input of the illumination attention module, *S*() is the spatial attention module, *C*() represents the channel attention module. The channel attention module *C*() can be expressed as:

2 $$\begin{aligned} C(x) = x \times \delta \left\{ \begin{array}{l} Conv_1 \left[ {Avg(x)} \right] \oplus Conv_1 \left[ {Max(x)} \right] \\ \oplus Conv_1 \left[ {Max(x) \oplus Avg(x)} \right] \\ \end{array} \right\} \end{aligned}$$

where $\delta ()$ represents the Sigmoid function, *Max*() and *Avg*() represent the maximum pooling operation and the average pooling operation, respectively, represents the sum of elements, and $Conv_1 ()$ represents an independent convolution layer.

The spatial attention *S*() can be expressed as:

3 $$\begin{aligned} S(y) = y \times \delta \left\{ {Conv_2 \left[ {Max(y)[]Avg(y)} \right] } \right\} \end{aligned}$$

where *y* is the output of the channel attention module, i.e. $y = C(x)$. [] is the concatenation operation. $Conv_2 ()$ is the parallel convolution layer, which can be expressed as:

4 $$\begin{aligned} \begin{array}{c} Conv_2 (x) = Conv[Conv_{1 \times 1} (x)] \oplus Conv[Conv_{3 \times 3} (x)] \oplus Conv[Conv_{5 \times 5} (x)] \\ \end{array} \end{aligned}$$

where $Conv_{1 \times 1} ()$, $Conv_{3 \times 3} ()$, $Conv_{5 \times 5} ()$ represent the standard convolution with LeakyReLU activation function with convolution kernels of 1, 3, and 5, respectively.

#### Proposed adversarial network

**Figure 6:**
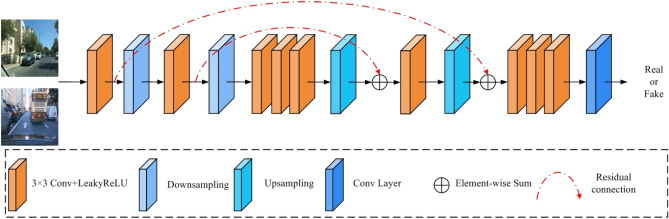
Our proposed forward adversarial network.

Our proposed generative adversarial network comprises both forward and reverse adversarial networks with the same structure. Therefore, we will only introduce the forward adversarial network in this context. The multiscale adversarial network with the Markovian structure we designed is illustrated in Fig. 6. Our method differs from standard adversarial networks in using convolutional layers instead of fully connected ones for feature extraction. This leads to a reduction in the number of model parameters. Our approach involves two downsampling operations and two upsampling operations. Downsampling is used to reduce the size of the feature map, which expands the receptive field. On the other hand, upsampling restores the feature map, enhancing the adversarial network’s overall discriminative capacity. Additionally, we incorporate two skip connections to prevent information loss and improve the discriminative ability of the adversarial network.

#### Proposed loss function

We proposed the loss function of our designed generative adversarial network based on the loss function of CycleGAN. We introduce the unsupervised perceptual loss and use the least squares function instead of the cross-entropy function in the original adversarial loss. Our total loss function can be expressed as follows:

5 $$\begin{aligned} \begin{array}{c} L(G_{X \rightarrow Y} ,G_{Y \rightarrow X} ,D_{X \rightarrow Y} ,D_{Y \rightarrow X} ) = L_{Adv} (G_{X \rightarrow Y} ,D_{X \rightarrow Y} ,X,Y) + L_{Adv} (G_{Y \rightarrow X} ,D_{Y \rightarrow X} ,X,Y)+ \\ L_{Cyc} (G_{X \rightarrow Y} ,G_{Y \rightarrow X} ) + L_{Identity} (G_{X \rightarrow Y} ,G_{Y \rightarrow X} ) + L_{Perceptual} (G_{X \rightarrow Y} ,G_{Y \rightarrow X} ) \\ \end{array} \end{aligned}$$

where $$L_{Adv} $$ is the adversarial loss, $$L_{Cyc} $$ is the cycle-consistency loss, $$L_{identity} $$ is the identity loss and $$L_{perceptual} $$ is the unsupervised perceptual loss. $$G_{X \rightarrow Y} $$, $$D_{X \rightarrow Y} $$, $$G_{Y \rightarrow X} $$ and $$D_{Y \rightarrow X} $$ represent the forward generative network, forward discriminative network, reverse generative network and reverse discriminative network, respectively. The adversarial loss $$L_{Adv} $$ can be expressed as follows:

6 $$\begin{aligned} L_{Adv} (G_{X \rightarrow Y},D_{X \rightarrow Y},X,Y)= & {} E_{y\sim p_{data(y)} } \left[ {\left\| {D_{X \rightarrow Y} (y) - 1} \right\| _2 } \right] + E_{x\sim p_{data(x)} } \left[ {\left\| {D_{X \rightarrow Y} (G_{X \rightarrow Y} (x))} \right\| _2 } \right] \end{aligned}$$

7 $$\begin{aligned} L_{Adv} (G_{Y \rightarrow X},D_{Y \rightarrow X},X,Y)= & {} E_{x\sim p_{data(x)} } \left[ {\left\| {D_{Y \rightarrow X} (x) - 1} \right\| _2 } \right] + E_{y\sim p_{data(y)} } \left[ {\left\| {D_{Y \rightarrow X} (G_{Y \rightarrow X} (y))} \right\| _2 } \right] \end{aligned}$$

where *Y* and *X* represent the high-quality image domain and low-quality image domain, respectively. $$P_{data(x)} $$ and $$P_{data(y)} $$ represent the nighttime road scene image training samples and normal road scene image training samples.

The cycle-consistency loss $$L_{Cyc} $$ is used to reduce the distance between the reconstructed image and the original image. The cycle-consistency loss can be expressed as follows:

8 $$\begin{aligned} L_{Cyc} (G_{X \rightarrow Y} ,G_{Y \rightarrow X} ) = E_{\sim p_{data(x)} } \left[ {\left\| {G_{Y \rightarrow X} (G_{X \rightarrow Y} (x)) - x} \right\| _1 } \right] + E_{y\sim p_{data(y)} } \left[ {\left\| {G_{X \rightarrow Y} (G_{Y \rightarrow X} (y)) - y} \right\| _1 } \right] \end{aligned}$$

The identity loss $$L_{perceptual} $$ is used to maintain the content and style of the original image. The identity loss can be expressed as follows:

9 $$\begin{aligned} L_{Identity} (G_{X \rightarrow Y} ,G_{Y \rightarrow X} ) = E_{x\sim p_{data(x)} } \left[ {\left\| {G_{X \rightarrow Y} (x) - x} \right\| _1 } \right] + E_{y\sim p_{data(y)} } \left[ {\left\| {G_{Y \rightarrow X} (y) - y} \right\| _1 } \right] \end{aligned}$$

Unsupervised perceptual loss aims to reduce the overarching disparity between the generative network’s input and output images. The equation for perceptual loss is described as:

10 $$\begin{aligned} L_{Perceptual} (G_{X \rightarrow Y} ,G_{Y \rightarrow X} ) = \frac{1}{{WH}}\sum \limits _{i = 1}^W {\sum \limits _{j = 1}^H {||\varphi (G_{X \rightarrow Y} (x)) - \varphi (x)||_2 } } + \frac{1}{{WH}}\sum \limits _{i = 1}^W {\sum \limits _{j = 1}^H {||\varphi (G_{Y \rightarrow X} (y)) - \varphi (y)||_2 } } \end{aligned}$$

where *W* and *H* represent the width and height of the feature map, respectively. $\varphi ()$ is the feature maps extracted by the first convolution layer after the 5th pooling layer in the pre-trained VGG16 model.

## Simulation and discussion

We evaluate the efficacy of various methods utilizing the BDD100K dataset^39^. We conduct a comprehensive comparison between our proposed approach and four alternative methods: CycleGAN^24^, Uretinex-Net^6^, EnlightenGAN^35^, Zero-DCE^23^, Zero-DCE++^15^, and LumiNet^28^. To assess the performance quantitatively, we use a set of image quality evaluation metrics, including PSNR^40^, SSIM^41^, NIQE^42^, and MetaIQA^43^. Larger values of PSNR, SSIM, MetaIQA, and smaller NIQE values correspond to images of higher quality. We set the batch size to 16 in our experimental process and initialized all other auxiliary parameters to 0. To ensure the fairness of the experiment, we used the same Adam optimizer with a batch size of 16, and parameters ($\beta _1 = 0.5$, $\beta _2 = 0.999$ are used to adjust the decay rate of the exponential moving average) to optimize the network’s loss function. All networks were trained from scratch with a learning rate of 0.0001. We keep the same learning rate for the first 100 epochs and linearly decay the rate to zero over the next 100 epochs. The EnlightenGAN and CycleGAN all used the Adam optimizer to optimize the networks. We also use CycleGAN’s training method to train all networks. They replace the negative log-likelihood objective in the loss function with a least square loss. This loss performs more stably during training and generates higher-quality results. The operating system is Ubuntu 18.04, and the GPU is NVIDIA GTX 2080Ti.

### Datasets and metrics

**Figure 7:**
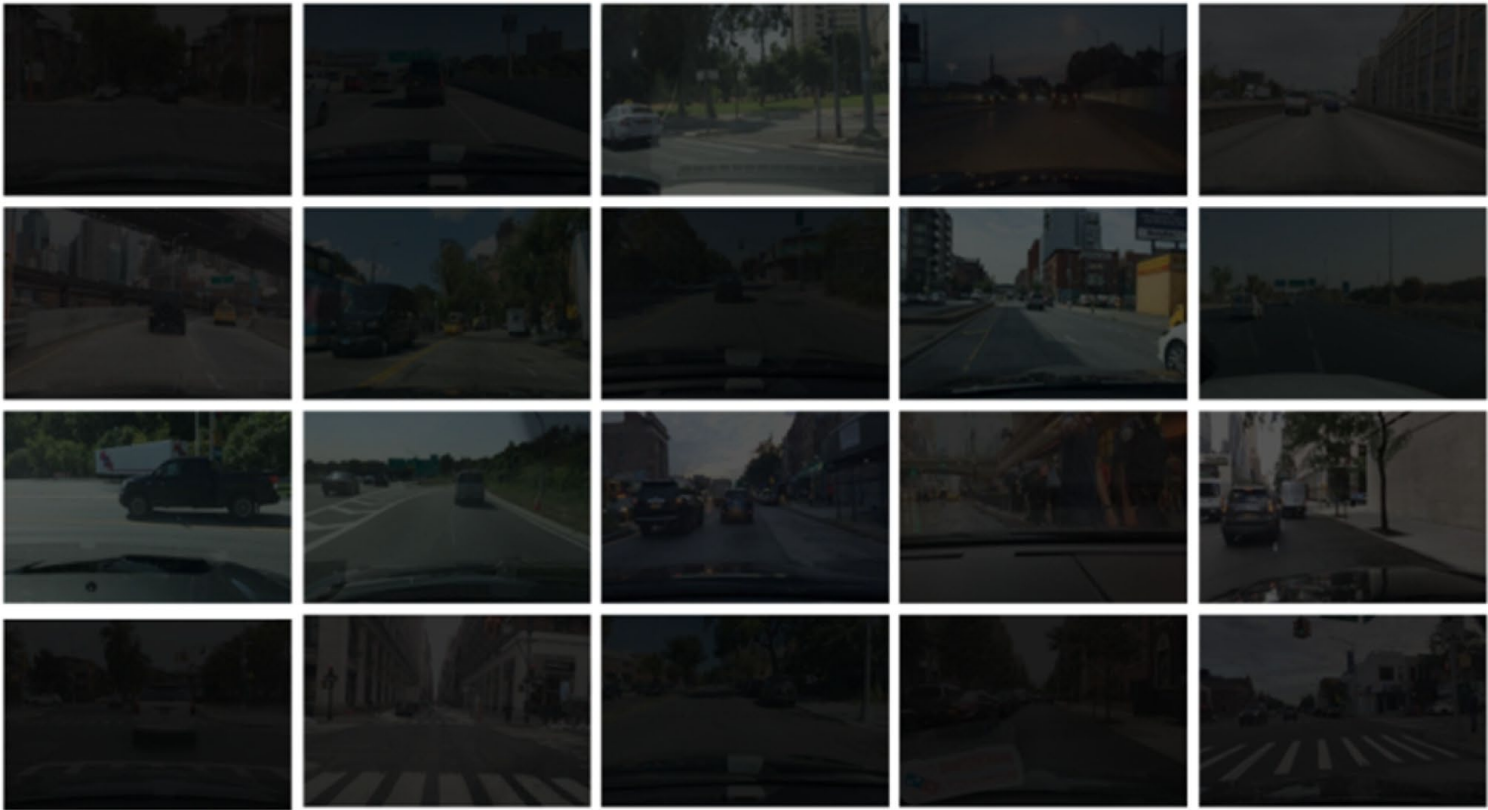
The partial synthetic night road scene images for training.

To demonstrate the effectiveness of our proposed method, we carried out simulation tests using the BDD100K dataset^39^ for autonomous driving. We select 2000 images of normal illumination road scenes in different scenes from the BDD100K dataset. We use 1000 images to synthesize road scene images in an extremely dark environment. We synthesize the low-illumination images using the exponential transformation method. It can be expressed as:

11 $$\begin{aligned} I_{out} = \alpha \cdot I_{in}^\gamma \end{aligned}$$

where $$\alpha $$ is the illumination scaling factor. $$I_{in} $$ is the input image and $$I_{out} $$ is the generated nighttime low illumination image. $$\gamma $$ is the illumination scaling index. We set the illumination scaling index to [0.6, 0.8]. The partial synthetic night road scene images for training are shown in Fig. 7. We used 1000 normal illumination road scene images, 800 synthetic road scene images as the training set, and 200 synthetic low-illumination road scene images as the test set.

### Simulation on synthetic dataset

**Figure 8:**
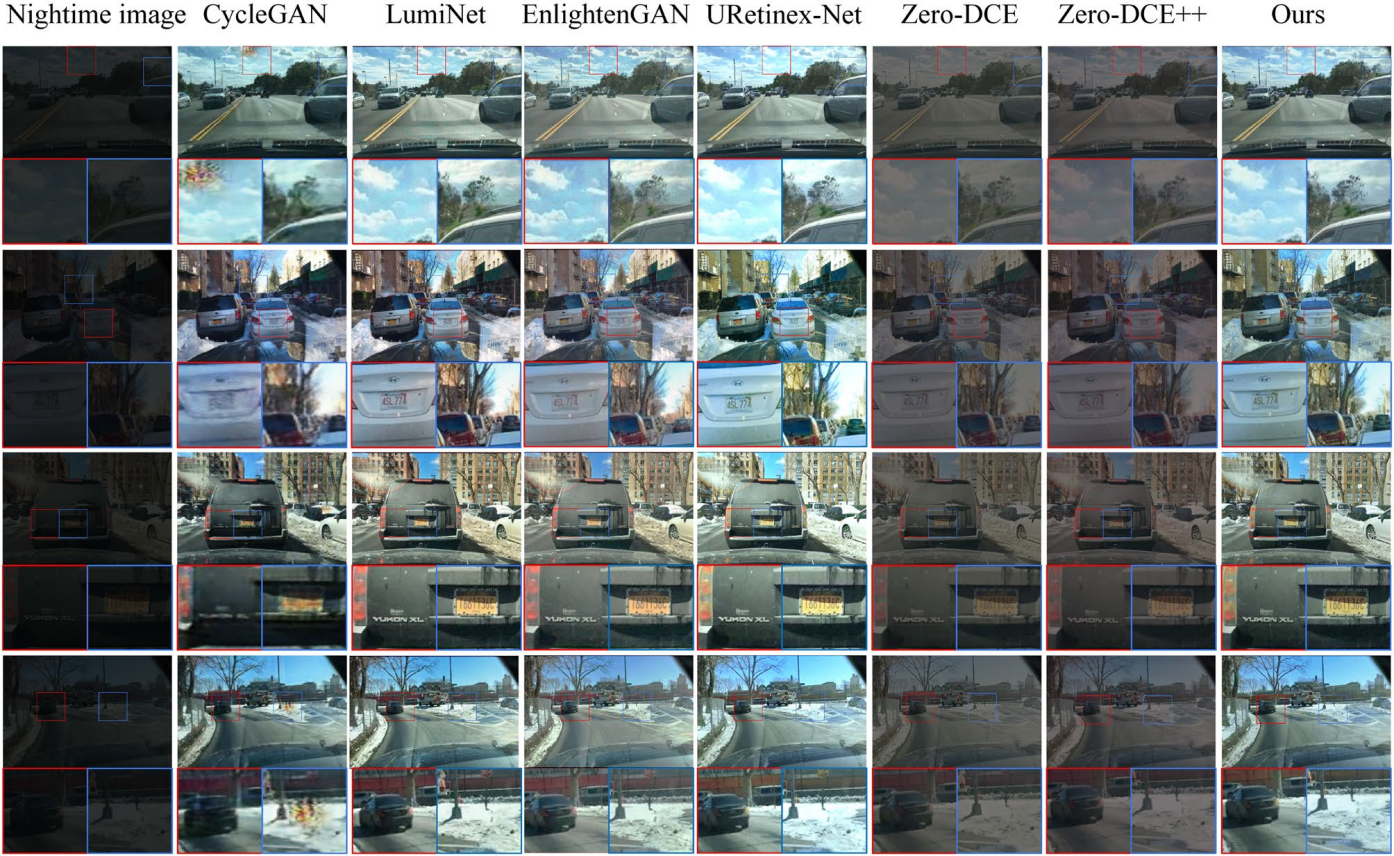
Image enhancement results of each method.

We selected four images randomly from the test dataset for input and compared our proposed method and other approaches. The resulting enhanced images are displayed in Fig. 8. The first column in Fig. 8 represents the input low-quality nighttime road scene image. Subsequent columns, from the second to the eighth, display images enhanced by the CycleGAN, LumiNet, EnlightenGAN, Uretinex-Net, Zero-DCE method, Zero-DCE++ method, and our proposed method, respectively. It is evident that both the Zero-DCE and Zero-DCE++ methods result in underexposed images. Enhanced images by the CycleGAN method appear noisy in the local enlarged portion area, leading to blurriness. The local brightness levels produced by the URetinex-Net are excessively high for the cloud image in the first row, the building image in the second row, and the snow image in the fourth row. Color distortions exist in the snow and license plate images of EnlightenGAN and LumiNet in the third and fourth-row images. Although the images produced by our proposed method exhibit some distortion in the snow, it is relatively minor.

**Figure 9:**
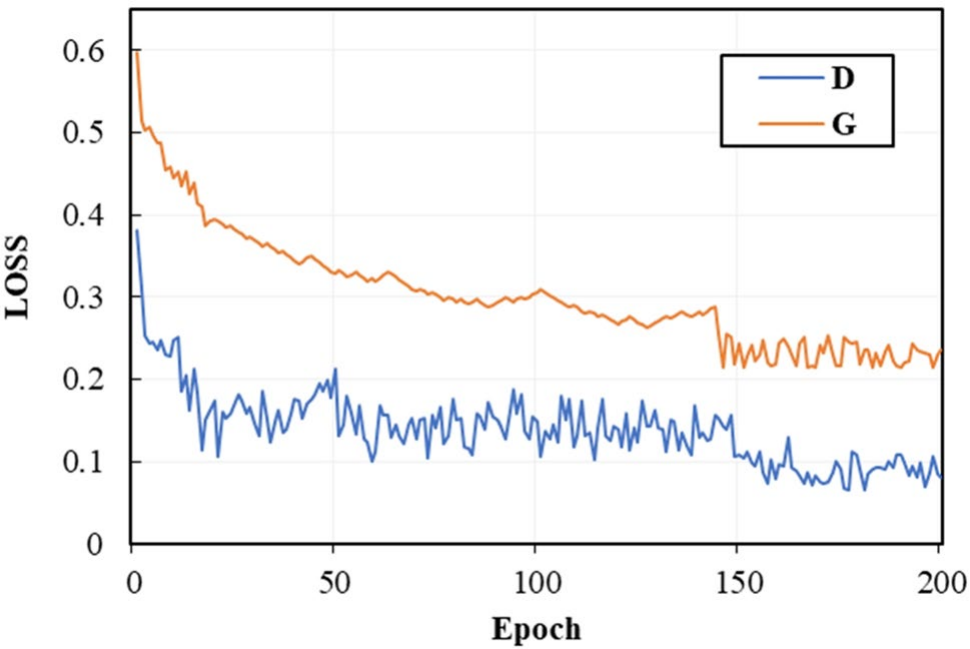
Convergence curve of the proposed Generator and Discriminator(D:Discriminator;G:Generator).

To quantitatively analyze the performance of the methods, we utilize four evaluation metrics: PSNR, SSIM, NIQE, and MetaIQA to measure the quality of enhanced images. The test dataset contains 200 randomly selected synthetic nighttime road scene images. The outcomes are presented in Table 1. Our proposed method has the largest PSNR, SSIM, and MetaIQA values and the smallest NIQE value. This indicates that the enhanced image quality obtained by our proposed method is the highest. It also shows that the proposed method exhibits better capability in enhancing nighttime road images than other methods. We also tested the computational complexity and running time of the different methods. The test results are presented in Table 2. The CycleGAN has the largest Floating Point Operations(FLOPs), followed by our proposed method. The running time of CycleGAN is also the longest, followed by our proposed method. To extract rich feature information and achieve better enhancement of nighttime road scene images, our designed model is complex, leading to higher computational complexity and slower image processing speed. The convergence curves of the proposed generator and discriminator are shown in Fig. 9. The two curves start to converge around 150 epochs.

**Table 1:** Comparison of image enhancement performance in the synthetic road scene test dataset.

	CycleGAN	LumiNet	EnlightenGAN	Uretinex-Net	Zero-DCE	Zero-DCE++	Our method
PSNR	20.865	26.796	26.657	25.694	25.151	26.530	28.633
SSIM	0.793	0.856	0.842	0.806	0.790	0.829	0.863
NIQE	7.347	4.683	4.596	4.729	4.366	4.414	4.344
MetaIQA	0.242	0.388	0.368	0.355	0.342	0.352	0.403

**Table 2:** Floating point operations and running time.

	CycleGAN	LumiNet	EnlightenGAN	Uretinex-Net	Zero-DCE	Zero-DCE++	Our method
FLOPs (G)	217.44	185.52	107.90	172.52	38.02	4.84	198.10
Running times/image(s)	0.172	0.0680	0.0602	0.0654	0.0344	0.0106	0.0816

### Simulation on images with different illumination

**Figure 10:**
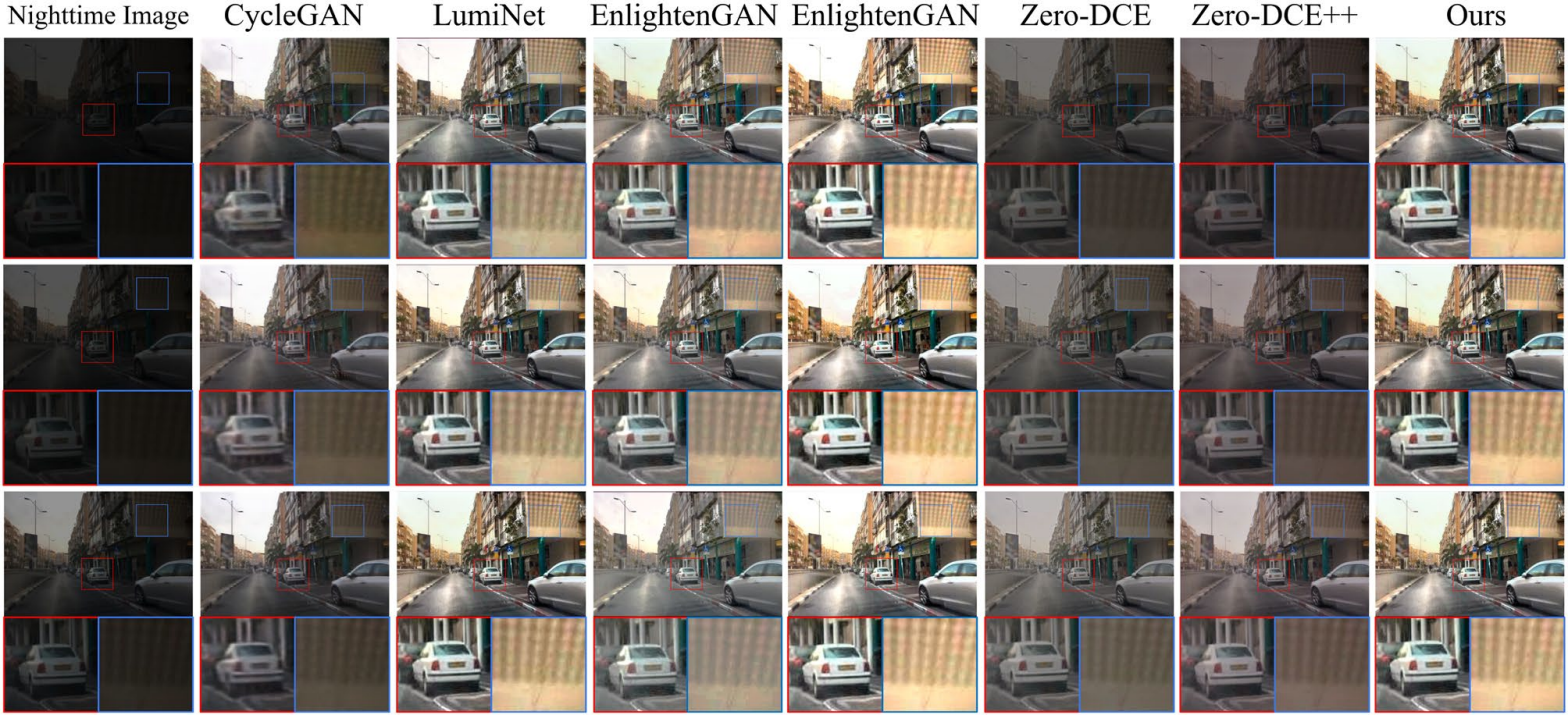
Image enhancement results of each method under different illumination intensities.

To comprehensively assess the image enhancement outcomes across varying illumination scenarios, we introduced illumination intensity variations by selecting an image from the dataset and adjusting the illuminance scaling factor to 0.7, 0.8, and 0.9. Figure 10 illustrates the enhanced images generated by different methods under these varied illumination intensities. As the illumination intensity decreases, the quality of the enhanced images gradually deteriorates. The images generated by CycleGAN, Zero-DCE, and Zero-DCE++ exhibit noticeable underexposure. We have compiled the PSNR, SSIM, NIQE, and MetaIQA values for various methods to provide a more quantified understanding in Table 3. Our proposed method achieves the largest PSNR, SSIM, and MetaIQA values while maintaining the smallest NIQE value. This indicates that our method outperforms others in enhancing nighttime road images.

**Table 3:** Comparison of image enhancement performance under different light intensities.

	Light intensities	CycleGAN	LumiNet	EnlightenGAN	Uretinex-Net	Zero-DCE	Zero-DCE++	Our
PSNR	0.7	21.881	24.562	23.899	24.526	23.312	23.301	26.351
PSNR	0.8	23.522	25.699	24.354	26.772	25.011	25.965	28.217
PSNR	0.9	24.013	27.253	25.998	27.954	26.998	26.087	28.063
PSNR	Average	23.139	25.838	24.750	26.417	25.107	25.118	27.544
SSIM	0.7	0.763	0.802	0.815	0.824	0.796	0.783	0.871
SSIM	0.8	0.681	0.813	0.862	0.869	0.722	0.792	0.903
SSIM	0.9	0.702	0.863	0.892	0.917	0.835	0.819	0.927
SSIM	Average	0.715	0.826	0.856	0.870	0.784	0.798	0.900
NIQE	0.7	6.797	3.780	3.814	3.798	5.175	5.182	3.772
NIQE	0.8	8.864	3.776	3.765	3.531	4.823	4.634	3.443
NIQE	0.9	9.837	3.730	3.678	3.390	4.722	4.623	3.344
NIQE	Average	8.499	3.762	3.752	3.573	4.907	4.813	3.520
MetaIQA	0.7	0.235	0.438	0.435	0.501	0.301	0.261	0.517
MetaIQA	0.8	0.251	0.415	0.487	0.498	0.338	0.308	0.532
MetaIQA	0.9	0.244	0.396	0.381	0.395	0.356	0.332	0.397
MetaIQA	Average	0.243	0.416	0.434	0.465	0.332	0.300	0.482

### Simulation on real nighttime road scene images

**Figure 11:**
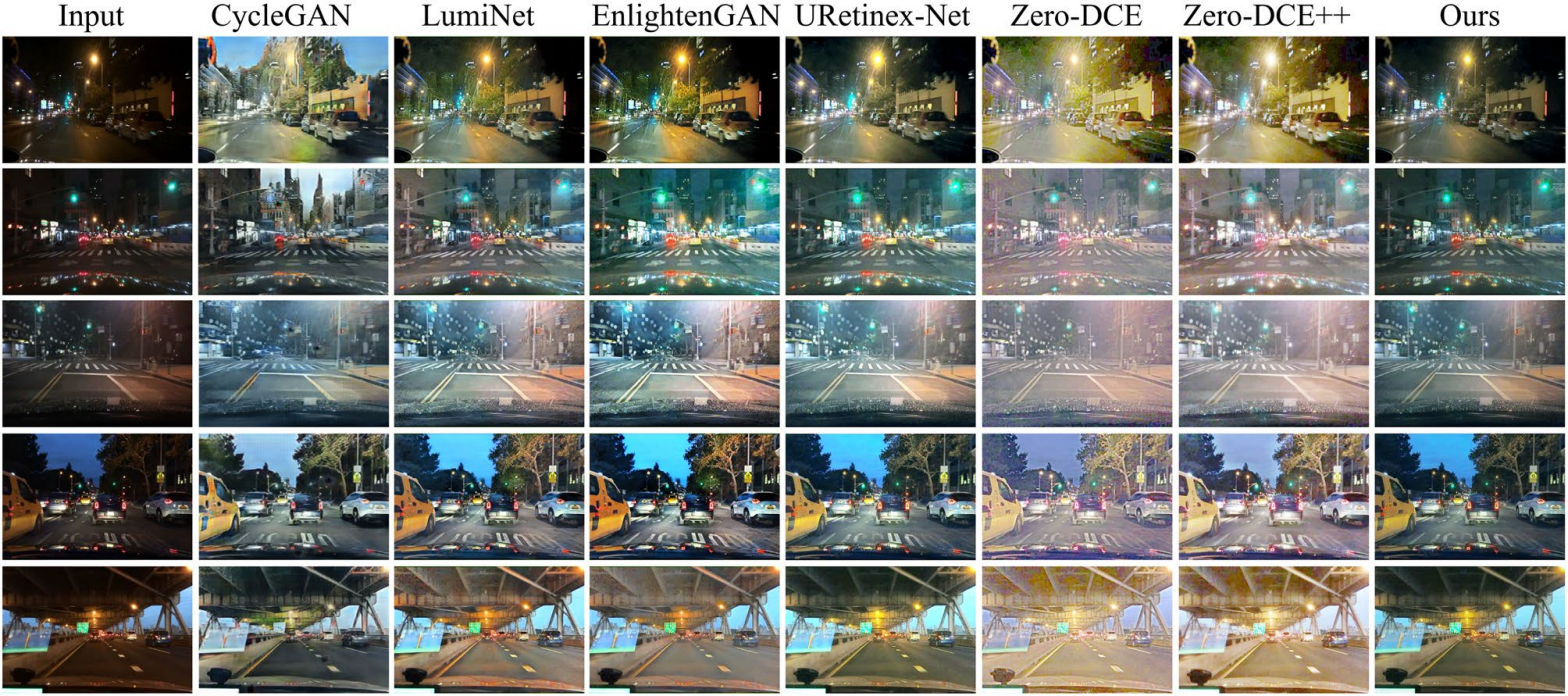
Real night road scene images selected from the BDD100K dataset and enhanced images by different methods.

We assess the performance of different methods for enhancing images using real nighttime road scene images from the BDD100K dataset. Figure 11 displays the enhanced images obtained by various methods. The first column shows the original image, while the following columns display the enhanced images produced by CycleGAN, LumiNet, EnlightenGAN, Uretinex-Net, Zero-DCE method, Zero-DCE++ method, and our method. In Fig. 11, it can be observed that all the images produced by the Zero-DCE method and the Zero-DCE++ method have overexposure. The first and second images generated by the CycleGAN Method exhibit significant distortion and deformation. The tree image in the upper right corner appears distorted in the fourth image generated by LumiNet and EnlightenGAN methods. In the third image generated by the EnlightenGAN method, the building in the upper left appears darker, while the building in the upper right appears brighter. This indicates that the contrast of the images enhanced by the EnlightenGAN method is excessively increased. The images generated by our method also have some distortion, but the distortion is relatively minor. To objectively assess the image quality depicted in Fig. 11, we utilize NIQE and MetalQA. The results are shown in Table 4. The proposed method generates images with the largest MetaIQA value and the smallest NIQE value. This indicates that images enhanced by our method possess higher quality than others.

**Figure 12:**
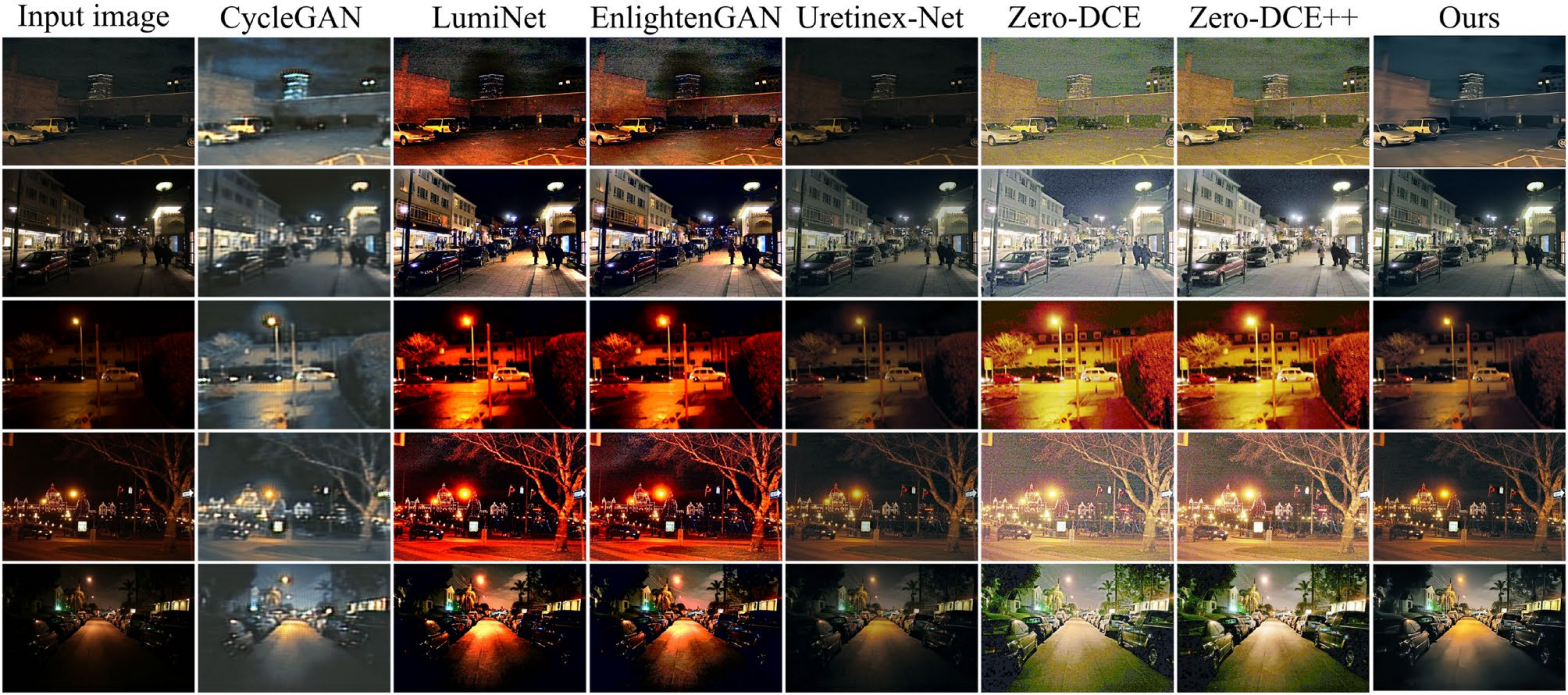
Real night road scene images selected from the ExDark dataset and enhanced images by different methods.

The ExDark dataset contains real low-light images. To further validate the algorithm on real images, we used images from the car, bus, and bicycle categories in the ExDark dataset as the test dataset. We selected five real low-light images from the test dataset to test the methods. The images are displayed in Fig. 12. The images produced by CycleGAN exhibit blurred details, while those generated by the LumiNet method display excessive contrast. Images generated by the EnlightenGAN method suffer from color distortion. Images generated by the Uretinex-Net method exhibit underexposure. Both the Zero-DCE and Zero-DCE++ methods result in overexposed images. Our proposed method effectively enhances the brightness and clarity of low-light images, resulting in more natural-looking enhanced images. In Fig. 12, the images suffer from insufficient brightness and complex backgrounds, making it difficult for some methods to extract adequate features. Consequently, the enhanced images exhibit color and detail distortions. However, Our method has strong feature extraction capabilities, allowing it to extract relatively more features. As a result, the distortions in the enhanced images of our method are relatively minimal. To quantitatively analyze the performance of the methods. We use test datasets to test the methods. The performances of different methods are shown in Table 5. Table 5 shows that our method has the smallest NIQE value and the largest MetaIQA value. This indicates that our method performs better in enhancing nighttime road images than other methods.

**Table 4:** Valuation metrics of enhanced images shown in Fig. 11.

Images	Valuation metrics	CycleGAN	LumiNet	EnlightenGAN	Uretinex-Net	Zero-DCE	Zero-DCE++	Our method
1st	NIQE	8.644	4.115	4.041	3.983	4.559	4.771	3.578
1st	MetaIQA	0.188	0.217	0.223	0.232	0.206	0.192	0.247
2nd	NIQE	7.010	3.643	3.903	3.802	4.215	4.203	3.415
2nd	MetaIQA	0.117	0.201	0.182	0.187	0.178	0.179	0.215
3rd	NIQE	5.772	3.809	3.789	3.787	5.464	5.079	3.468
3rd	MetaIQA	0.214	0.246	0.291	0.297	0.226	0.236	0.301
4th	NIQE	4.837	3.788	3.805	3.576	5.431	5.309	3.471
4th	MetaIQA	0.201	0.225	0.219	0.238	0.180	0.191	0.253
5th	NIQE	5.389	3.914	3.849	4.241	4.732	4.463	3.476
5th	MetaIQA	0.223	0.267	0.275	0.252	0.235	0.239	0.298

**Table 5:** Comparison of image enhancement performance in real night road scene images in Fig. 12.

	CycleGAN	LumiNet	EnlightenGAN	Uretinex-Net	Zero-DCE	Zero-DCE++	Our method
NIQE	5.158	3.958	3.903	3.865	4.038	4.152	3.514
MetaIQA	0.263	0.416	0.425	0.480	0.313	0.329	0.498

**Figure 13:**
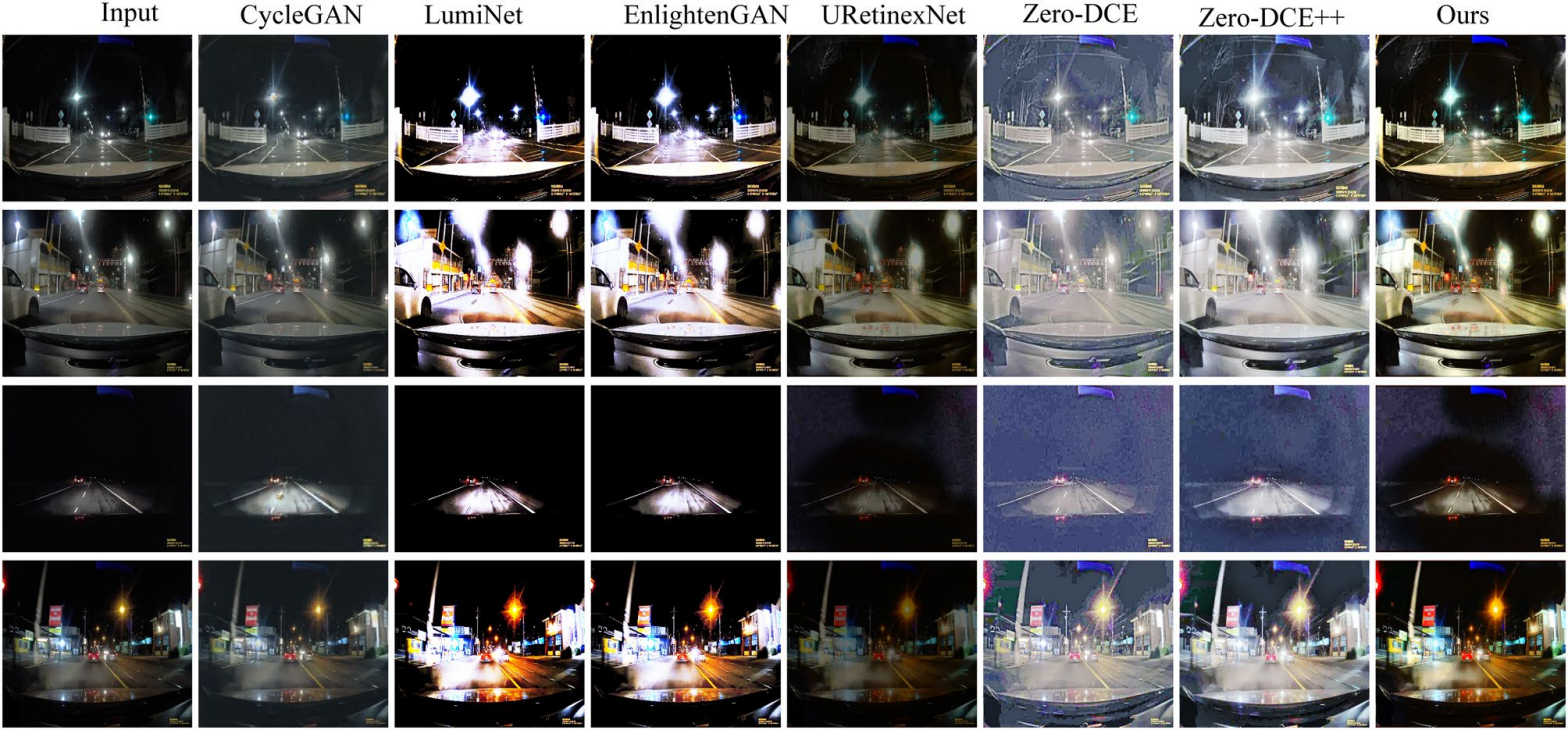
Real night road scene images selected from the nighttime object detection dataset and enhanced images by different methods.

To further validate the generalization performance of the proposed network, we conducted a comparative analysis on another nighttime road scene dataset (nighttime object detection dataset) for nighttime object detection. We randomly selected four low-light images from this dataset to test the image enhancement performance of each method. The experimental results are shown in Fig. 13. The brightness of the images enhanced by the CycleGAN method is the lowest, with the poorest quality. From the reflection of light on the ground, it can be observed that the images enhanced by the LumiNet and EnlightenGAN methods suffer from excessive enhancement in local bright areas. The images enhanced by the UretinexNet method exhibit underexposure issues. Overexposure phenomena are observed in the images enhanced by the Zero-DCE and Zero-DCE++ methods. Although the images enhanced by our proposed algorithm exhibit partial underexposure in the road areas, they appear more natural overall with better visual effects. To objectively evaluate the algorithm’s performance, we also utilized no-reference evaluation metrics NIQE and MetaIQA to assess image quality. The average results are shown in Table 6, indicating that the images enhanced by our proposed method have the lowest NIQE and the highest MetaIQA. This suggests that the images enhanced by our proposed algorithm are more natural and clear, further demonstrating the good generalization capability of our algorithm.

**Table 6:** Comparison of image enhancement performance in real night road scene images in Fig. 12.

	CycleGAN	LumiNet	EnlightenGAN	Uretinex-Net	Zero-DCE	Zero-DCE++	Our method
NIQE	7.653	4.944	5.084	4.771	4.978	4.785	4.721
MetaIQA	0.263	0.269	0.265	0.287	0.267	0.276	0.301

Our designed illumination attention module can more effectively adjust the brightness and contrast of images based on varying light intensities in nighttime road scenes. This effectively prevents overexposure, thus improving the visual quality of the images. Additionally, introducing the context feature extraction module allows our model to capture more contextual semantic information. This helps preserve important details such as road signs and vehicle contours during the image enhancement process, thereby enhancing the accuracy of road scene image recognition. Furthermore, the designed receptive field residual module effectively enhances the extraction of global image information, further improving image clarity and making details in nighttime road scenes more distinguishable. Moreover, by incorporating unsupervised perceptual loss and mean square loss functions, our method better maintains the authenticity of the images, avoiding over-enhancement and the appearance of artifacts, resulting in more natural-looking enhanced road scene images. Therefore, compared to other methods, the images enhanced by our method have less distortion and better clarity and visual appeal.

### Ablation study

Ablation studies are an essential research methodology used to evaluate the individual impact and significance of specific components or factors within a model. By systematically removing or modifying certain elements, researchers can gain insights into the importance of these components in the model’s overall performance. We validate the effectiveness of each proposed module by removing it from the complete network. If the network’s performance changes insignificantly after removing a module, it indicates that the module has a relatively minor impact. Conversely, if the performance changes significantly, it demonstrates that the module has a substantial effect. We perform ablation studies to validate every module’s efficacy within our proposed method. We conducted six experiments on the images with mixed illumination, which are the proposed method without the receptive field residual module (No_RFRM), the proposed method without the illumination attention module (No_IAM), the proposed method without the context feature extraction module (No_CFEM), the proposed method without the multiscale discriminative network (No_MSDN)(Using the discriminative network of cycleGAN instead of our discriminative network.), the proposed method without the unsupervised perceptual loss function (No_ULoss) and the proposed method without the least squares loss function (No_LSLoss). The more significant the performance gap between the proposed method without a proposed module and the complete method, the greater the impact of that proposed module. The results are shown in Table 7. Compared to the complete method, the methods No_RFRM, No_IAM, No_CFEM, No_MSDN, No_ULoss, and No_LSLoss all exhibit smaller PSNR, SSIM, and MetaIQE values and larger NIQE value. This shows that the performance decreases when the proposed modules are absent from the complete method. This also indicates that every module we’ve proposed plays a crucial role in enhancing nighttime road scene images. The No_CFEM method has the smallest PSNR, SSIM, and MetaIQE values and the largest NIQE than other methods. This indicates that compared to other proposed modules, the context feature extraction module has a greater impact on improving the performance of our method. Using the same analytical method, we can infer that compared to other proposed modules, the least squares loss function has the least impact on improving the performance of our method.

**Table 7:** Evaluation results for each module.

	No_RFRM	No_IAM	No_CFEM	No_MSDN	No_ULoss	No_LSLoss	Our method
PSNR	23.527	25.997	21.437	26.366	25.732	26.877	28.633
SSIM	0.638	0.782	0.631	0.815	0.763	0.823	0.863
NIQE	6.322	6.423	7.021	5.563	6.013	5.225	4.344
MetaIQA	0.254	0.268	0.229	0.384	0.267	0.359	0.403

## Conclusion

This paper introduces an innovative cycle-consistent generative adversarial network intended for enhancing nighttime road scene images. The generative network comprises the receptive field residual module, context feature extraction module, and illumination attention module. These, along with convolutional modules, contribute to constructing the encoder-decoder network. A multiscale discriminative network is proposed in the discriminative network to enhance its discriminative ability. Furthermore, a dedicated loss function is introduced for the novel cycle-consistent generative adversarial network. Our approach is assessed using synthetic nighttime road scene images, which perform better than other methods. This is demonstrated by the largest PSNR, SSIM, and MetaIQA values, as well as the smallest NIQE value, across varied illumination intensities and diverse synthetic images. Real nighttime road scene images are also employed for evaluation. Our method generated clearer and more natural-looking images. Additionally, the images generated by our method have the highest MetaIQA score and the lowest NIQE score. This suggests that our proposed method performs better in enhancing nighttime road scene images.

Although our proposed method performs better in nighttime image enhancement, it has a complex structure that consumes more time. In future work, we will consider reducing the model’s complexity to balance real-time processing and image enhancement effects. Additionally, although the enhanced images are closer to real images, minor distortions in restoring road lane colors occur due to the influence of the vehicle’s headlights. In future work, we will also consider further reducing these distortions and improving the quality of enhanced images.

## Data Availability

All data generated or analyzed during this study are included in this published article.
